# 3-Chloro-*N*-(4-meth­oxy­phen­yl)propanamide

**DOI:** 10.1107/S1600536811040682

**Published:** 2011-10-08

**Authors:** Richard Betz, Thomas Gerber, Eric Hosten, Maravanahalli S. Siddegowda, Hemmige S. Yathirajan

**Affiliations:** a Nelson Mandela Metropolitan University, Summerstrand Campus, Department of Chemistry, University Way, Summerstrand, PO Box 77000, Port Elizabeth 6031, South Africa; b University of Mysore, Department of Studies in Chemistry, Manasagangotri, Mysore 570 006, India

## Abstract

The title compound, C_10_H_12_ClNO_2_, is a halogenated derivative of a secondary amide bearing an aromatic substituent. The C(=O)—N(H)—C_ar_—C_ar_ torsion angle of −33.70 (18)° rules out the presence of resonance spanning the amide as well as the aromatic system. In the crystal, classical N—H⋯O hydrogen bonds, as well as C–H⋯O contacts connect the mol­ecules into chains propagating along the *a* axis.

## Related literature

For structural similarity of *N*-substituted 2-aryl­acetamides to the lateral chain of natural benzyl­penicillin, see: Mijin & Marinkovic (2006 ▶); Mijin *et al.* (2008 ▶). For the coordination abilities of amides, see: Wu *et al.* (2008 ▶, 2010 ▶). For related structures, see: Akkurt *et al.* (2010 ▶); Huang & Xu, (2006 ▶); Moreno-Fuquen *et al.* (2011 ▶); Praveen *et al.* (2011 ▶). For the crystal structure of another compound featuring C—H⋯O contacts, see: Betz *et al.* (2011 ▶). For graph-set analysis of hydrogen bonds, see: Etter *et al.* (1990 ▶); Bernstein *et al.* (1995 ▶).
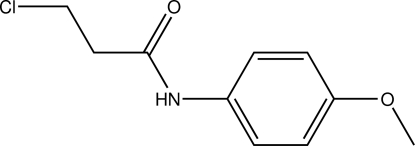


## Experimental

### 

#### Crystal data

C_10_H_12_ClNO_2_
*M* *_r_* = 213.66
Orthorhombic, 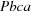

*a* = 9.6326 (3) Å
*b* = 8.6650 (2) Å
*c* = 25.7944 (8) Å
*V* = 2152.97 (11) Å^3^
*Z* = 8
Mo *K*α radiation
μ = 0.33 mm^−1^
*T* = 200 K
0.53 × 0.50 × 0.39 mm

#### Data collection

Bruker APEXII CCD diffractometer
Absorption correction: multi-scan (*SADABS*; Bruker, 2008 ▶) *T*_min_ = 0.921, *T*_max_ = 1.000
19180 measured reflections
2668 independent reflections
2401 reflections with *I* > 2σ(*I*)
*R*_int_ = 0.016

#### Refinement

*R*[*F* ^2^ > 2σ(*F* ^2^)] = 0.036
*wR*(*F* ^2^) = 0.096
*S* = 1.07
2668 reflections
132 parameters
H atoms treated by a mixture of independent and constrained refinement
Δρ_max_ = 0.28 e Å^−3^
Δρ_min_ = −0.33 e Å^−3^

### 

Data collection: *APEX2* (Bruker, 2010 ▶); cell refinement: *SAINT* (Bruker, 2010 ▶); data reduction: *SAINT*; program(s) used to solve structure: *SHELXS97* (Sheldrick, 2008 ▶); program(s) used to refine structure: *SHELXL97* (Sheldrick, 2008 ▶); molecular graphics: *ORTEP-3* (Farrugia, 1997 ▶) and *Mercury* (Macrae *et al.*, 2008 ▶); software used to prepare material for publication: *SHELXL97* and *PLATON* (Spek, 2009 ▶).

## Supplementary Material

Crystal structure: contains datablock(s) I, global. DOI: 10.1107/S1600536811040682/zj2028sup1.cif

Supplementary material file. DOI: 10.1107/S1600536811040682/zj2028Isup2.cdx

Structure factors: contains datablock(s) I. DOI: 10.1107/S1600536811040682/zj2028Isup3.hkl

Supplementary material file. DOI: 10.1107/S1600536811040682/zj2028Isup4.cml

Additional supplementary materials:  crystallographic information; 3D view; checkCIF report

## Figures and Tables

**Table 1:** Hydrogen-bond geometry (Å, °)

*D*—H⋯*A*	*D*—H	H⋯*A*	*D*⋯*A*	*D*—H⋯*A*
N1—H71⋯O1^i^	0.861 (18)	2.009 (18)	2.8643 (13)	172.1 (15)
C2—H2*B*⋯O1^i^	0.99	2.55	3.4203 (15)	147

Symmetry code: (i) 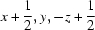.

**Fig. 1.**
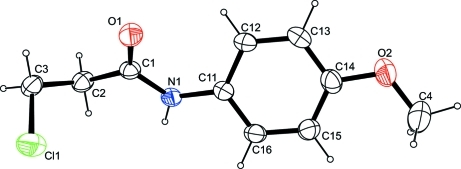
The molecular structure of the title compound, with atom labels and anisotropic displacement ellipsoids (drawn at 50% probability level).

**Fig. 2.**
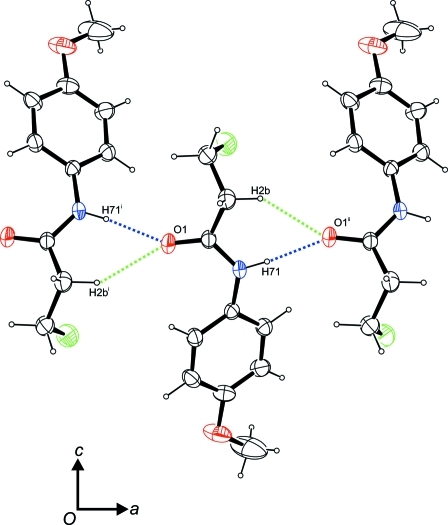
Intermolecular contacts, viewed along [0 - 1 0]. Blue dashed lines indicate classical hydrogen bonds of the N–H···O type, green dashed lines indicate C–H···O contacts. Symmetry operators: ix - 1/2, y, -z + 1/2; iix + 1/2, y, -z + 1/2.

**Fig. 3.**
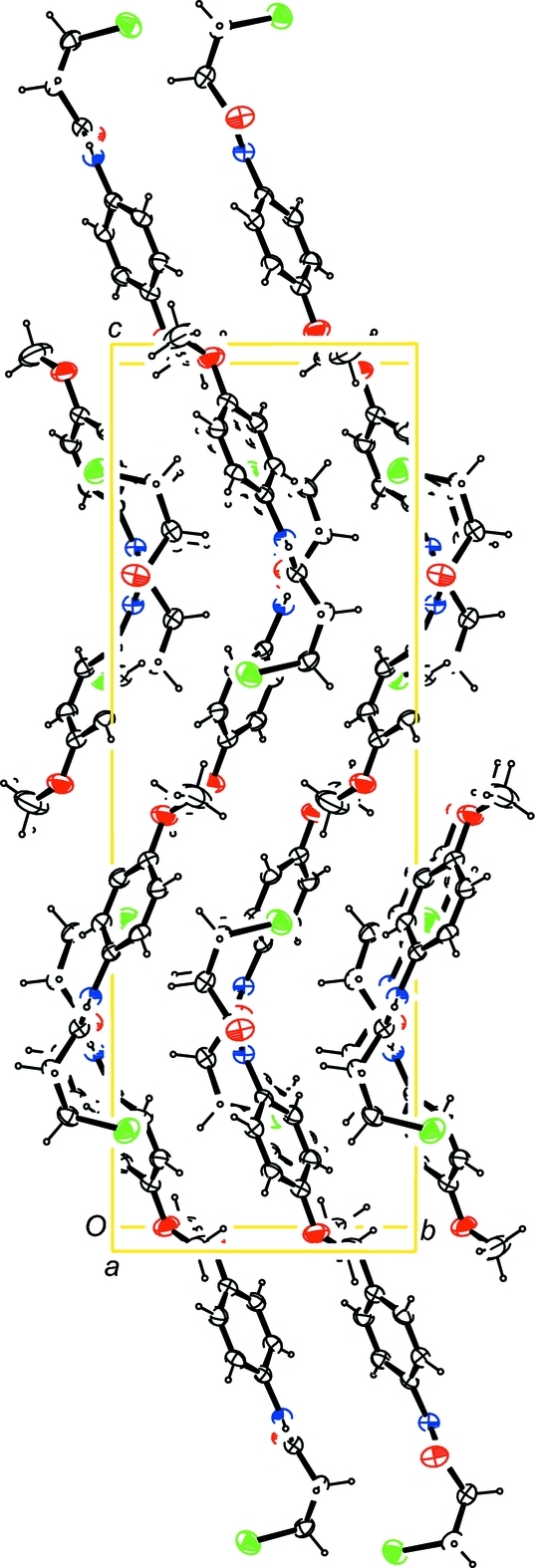
Molecular packing of the title compound, viewed along [-1 0 0] (anisotropic displacement ellipsoids drawn at 50% probability level).

## supplementary crystallographic information

### Comment

Amides are pervasive in nature and technology as structural materials. Amides are also used as ligands due to their excellent coordination abilities (Wu *et al.*, 2008; 2010). *N*-Substituted 2-arylacetamides are very interesting compounds because of their structural similarity to the lateral chain of natural benzylpenicillin (Mijin *et al.*, 2008; Mijin & Marinkovic, 2006). The crystal structure studies of some closely related compounds, *viz*., *N*-(2-bromophenyl)-2-phenylpropanamide (Huang & Xu, 2006), 3-chloro-*N*-(4-sulfamoylphenyl) propanamide (Akkurt *et al.*, 2010), 2-bromo-2-methyl-*N*-(4-nitrophenyl)propanamide (Moreno-Fuquen *et al.*, 2011) and *N*-(3-chloro-4-fluorophenyl)-2-(naphthalen-1-yl)acetamide (Praveen *et al.*, 2011) have been reported. Recently, the crystal structure of another compound featuring C–H···O contacts has been published (Betz *et al.*, 2011). In view of the importance of amides, the crystal structure of the title compound is reported.

The C=O bond length as well as the C(=O)—N(H) bond length of 1.2326 (14) Å and 1.3416 (15) Å, respectively, are indicative of typical amide-type resonance. The aromatic system of the phenyl moiety bonded to the amide's nitrogen atom, on the other hand, does not participate in this resonance as becomes apparent by the corresponding C(=O)—N(H)—C_ar_—C_ar_ dihedral angle of -33.70 (18) ° (Fig. 1).

In the crystal, classical hydrogen bonds of the N–H···O type as well as C–H···O contacts whose range falls by more than 0.1 Å below the sum of van-der-Waals radii of the atoms participating are observed in form of two homodromic chains. The C–H···O contacts stem from the methylene group adjacent to the C=O group and – in combination with the nitrogen-bond hydrogen atom – chelate the oxygen atom of the amide functionality. In terms of graph-set analysis (Etter *et al.*, 1990; Bernstein *et al.*, 1995), the descriptor for the classical hydrogen bonds as well as the C–H···O contacts is each *C*^1^_1_(4) on the unitary level. In total, the molecules are connected to chains along the crystallographic *a* axis. The shortest intercentroid distance between two centers of gravity was found at 4.8194 (8) Å (Fig. 2).

The packing of the title compound in the crystal is shown in Figure 3.

### Experimental

The title compound was obtained from *R. L*. Fine Chem., Bengaluru, India. X-ray quality crystals were obtained from dichloromethane by slow evaporation at room temperature.

### Refinement

Carbon-bound H atoms were placed in calculated positions (C—H 0.95 Å for aromatic carbon atoms and C—H 0.99 Å for methylene groups) and were included in the refinement in the riding model approximation, with *U*(H) set to 1.2*U*_eq_(C). The H atoms of the methyl group were allowed to rotate with a fixed angle around the C—C bond to best fit the experimental electron density (HFIX 137 in the *SHELX* program suite (Sheldrick, 2008), with *U*(H) set to 1.5*U*_eq_(C). The nitrogen-bound H atom was located on a difference Fourier map and refined freely.

### Crystal data

**Table:**

C_10_H_12_ClNO_2_	*D*_x_ = 1.318 Mg m^−^^3^
*M**_r_* = 213.66	Melting point = 388–391 K
Orthorhombic, *P**b**c**a*	Mo *K*α radiation, λ = 0.71073 Å
Hall symbol: -P 2ac 2ab	Cell parameters from 9900 reflections
*a* = 9.6326 (3) Å	θ = 2.4–28.3°
*b* = 8.6650 (2) Å	µ = 0.33 mm^−^^1^
*c* = 25.7944 (8) Å	*T* = 200 K
*V* = 2152.97 (11) Å^3^	Block, violet
*Z* = 8	0.53 × 0.50 × 0.39 mm
*F*(000) = 896	

### Data collection

**Table:**

Bruker APEXII CCD diffractometer	2668 independent reflections
Radiation source: fine-focus sealed tube	2401 reflections with *I* > 2σ(*I*)
graphite	*R*_int_ = 0.016
φ and ω scans	θ_max_ = 28.3°, θ_min_ = 2.6°
Absorption correction: multi-scan (*SADABS*; Bruker, 2008)	*h* = −11→12
*T*_min_ = 0.921, *T*_max_ = 1.000	*k* = −11→8
19180 measured reflections	*l* = −34→34

### Refinement

**Table:**

Refinement on *F*^2^	Primary atom site location: structure-invariant direct methods
Least-squares matrix: full	Secondary atom site location: difference Fourier map
*R*[*F*^2^ > 2σ(*F*^2^)] = 0.036	Hydrogen site location: inferred from neighbouring sites
*wR*(*F*^2^) = 0.096	H atoms treated by a mixture of independent and constrained refinement
*S* = 1.07	*w* = 1/[σ^2^(*F*_o_^2^) + (0.0406*P*)^2^ + 0.8819*P*] where *P* = (*F*_o_^2^ + 2*F*_c_^2^)/3
2668 reflections	(Δ/σ)_max_ = 0.001
132 parameters	Δρ_max_ = 0.28 e Å^−^^3^
0 restraints	Δρ_min_ = −0.33 e Å^−^^3^

### Fractional atomic coordinates and isotropic or equivalent isotropic displacement parameters (Å^2^)

**Table:**

	*x*	*y*	*z*	*U*_iso_*/*U*_eq_	
Cl1	0.63232 (4)	0.05020 (5)	0.363132 (15)	0.05120 (13)	
O1	0.45550 (9)	−0.07866 (13)	0.24620 (4)	0.0401 (2)	
O2	0.60693 (14)	0.17364 (14)	0.01725 (4)	0.0571 (3)	
N1	0.67622 (10)	−0.06661 (12)	0.21589 (4)	0.0278 (2)	
H71	0.7614 (19)	−0.0778 (18)	0.2253 (6)	0.039 (4)*	
C1	0.58029 (12)	−0.10651 (14)	0.25114 (5)	0.0277 (2)	
C2	0.63650 (13)	−0.19487 (15)	0.29700 (5)	0.0333 (3)	
H2A	0.6160	−0.3059	0.2922	0.040*	
H2B	0.7387	−0.1828	0.2980	0.040*	
C3	0.57679 (14)	−0.14268 (16)	0.34808 (5)	0.0359 (3)	
H3A	0.6073	−0.2138	0.3759	0.043*	
H3B	0.4742	−0.1460	0.3464	0.043*	
C4	0.7094 (3)	0.2727 (3)	−0.00413 (8)	0.0945 (9)	
H4A	0.7185	0.3650	0.0176	0.142*	
H4B	0.6819	0.3030	−0.0393	0.142*	
H4C	0.7986	0.2184	−0.0055	0.142*	
C11	0.65289 (11)	0.00051 (13)	0.16640 (4)	0.0257 (2)	
C12	0.53648 (12)	−0.03409 (14)	0.13633 (5)	0.0301 (2)	
H12	0.4654	−0.0985	0.1498	0.036*	
C13	0.52506 (13)	0.02579 (15)	0.08680 (5)	0.0344 (3)	
H13	0.4458	0.0022	0.0664	0.041*	
C14	0.62854 (15)	0.12028 (15)	0.06655 (5)	0.0367 (3)	
C15	0.74430 (15)	0.15511 (16)	0.09623 (5)	0.0368 (3)	
H15	0.8154	0.2194	0.0827	0.044*	
C16	0.75527 (13)	0.09486 (15)	0.14610 (5)	0.0315 (3)	
H16	0.8344	0.1189	0.1665	0.038*	

### Atomic displacement parameters (Å^2^)

**Table:**

	*U*^11^	*U*^22^	*U*^33^	*U*^12^	*U*^13^	*U*^23^
Cl1	0.0490 (2)	0.0532 (2)	0.0513 (2)	−0.01414 (17)	−0.00088 (16)	−0.01065 (16)
O1	0.0182 (4)	0.0644 (6)	0.0378 (5)	0.0037 (4)	0.0018 (3)	0.0059 (5)
O2	0.0822 (8)	0.0580 (7)	0.0312 (5)	−0.0186 (6)	−0.0137 (5)	0.0072 (5)
N1	0.0168 (4)	0.0368 (5)	0.0298 (5)	0.0016 (4)	−0.0016 (4)	0.0002 (4)
C1	0.0203 (5)	0.0319 (5)	0.0308 (5)	−0.0004 (4)	0.0000 (4)	−0.0018 (5)
C2	0.0274 (6)	0.0336 (6)	0.0389 (6)	0.0003 (5)	−0.0003 (5)	0.0068 (5)
C3	0.0330 (6)	0.0398 (7)	0.0350 (6)	−0.0080 (5)	0.0002 (5)	0.0094 (5)
C4	0.141 (2)	0.0964 (17)	0.0463 (10)	−0.0596 (17)	−0.0198 (12)	0.0292 (11)
C11	0.0219 (5)	0.0283 (5)	0.0268 (5)	0.0030 (4)	0.0009 (4)	−0.0046 (4)
C12	0.0240 (5)	0.0326 (6)	0.0337 (6)	−0.0012 (4)	−0.0024 (4)	−0.0044 (5)
C13	0.0331 (6)	0.0376 (6)	0.0324 (6)	0.0000 (5)	−0.0089 (5)	−0.0066 (5)
C14	0.0501 (8)	0.0333 (6)	0.0268 (5)	−0.0015 (6)	−0.0033 (5)	−0.0043 (5)
C15	0.0411 (7)	0.0376 (7)	0.0318 (6)	−0.0104 (6)	0.0034 (5)	−0.0030 (5)
C16	0.0266 (5)	0.0364 (6)	0.0315 (5)	−0.0051 (5)	−0.0003 (5)	−0.0058 (5)

### Geometric parameters (Å, °)

**Table:**

Cl1—C3	1.7973 (14)	C4—H4A	0.9800
O1—C1	1.2326 (14)	C4—H4B	0.9800
O2—C14	1.3690 (16)	C4—H4C	0.9800
O2—C4	1.419 (2)	C11—C16	1.3838 (17)
N1—C1	1.3416 (15)	C11—C12	1.3961 (16)
N1—C11	1.4207 (15)	C12—C13	1.3832 (18)
N1—H71	0.861 (18)	C12—H12	0.9500
C1—C2	1.5096 (17)	C13—C14	1.3917 (19)
C2—C3	1.5070 (18)	C13—H13	0.9500
C2—H2A	0.9900	C14—C15	1.3858 (19)
C2—H2B	0.9900	C15—C16	1.3923 (18)
C3—H3A	0.9900	C15—H15	0.9500
C3—H3B	0.9900	C16—H16	0.9500
			
C14—O2—C4	117.35 (13)	O2—C4—H4C	109.5
C1—N1—C11	127.26 (10)	H4A—C4—H4C	109.5
C1—N1—H71	115.8 (11)	H4B—C4—H4C	109.5
C11—N1—H71	116.8 (11)	C16—C11—C12	119.28 (11)
O1—C1—N1	123.45 (11)	C16—C11—N1	117.98 (10)
O1—C1—C2	122.01 (11)	C12—C11—N1	122.57 (11)
N1—C1—C2	114.50 (10)	C13—C12—C11	119.76 (11)
C3—C2—C1	113.33 (10)	C13—C12—H12	120.1
C3—C2—H2A	108.9	C11—C12—H12	120.1
C1—C2—H2A	108.9	C12—C13—C14	120.69 (11)
C3—C2—H2B	108.9	C12—C13—H13	119.7
C1—C2—H2B	108.9	C14—C13—H13	119.7
H2A—C2—H2B	107.7	O2—C14—C15	124.19 (13)
C2—C3—Cl1	110.75 (9)	O2—C14—C13	116.00 (12)
C2—C3—H3A	109.5	C15—C14—C13	119.80 (12)
Cl1—C3—H3A	109.5	C14—C15—C16	119.33 (12)
C2—C3—H3B	109.5	C14—C15—H15	120.3
Cl1—C3—H3B	109.5	C16—C15—H15	120.3
H3A—C3—H3B	108.1	C11—C16—C15	121.13 (11)
O2—C4—H4A	109.5	C11—C16—H16	119.4
O2—C4—H4B	109.5	C15—C16—H16	119.4
H4A—C4—H4B	109.5		
			
C11—N1—C1—O1	−5.5 (2)	C4—O2—C14—C15	−0.9 (2)
C11—N1—C1—C2	172.15 (11)	C4—O2—C14—C13	178.85 (18)
O1—C1—C2—C3	−45.01 (17)	C12—C13—C14—O2	−179.87 (12)
N1—C1—C2—C3	137.26 (11)	C12—C13—C14—C15	−0.1 (2)
C1—C2—C3—Cl1	−66.96 (12)	O2—C14—C15—C16	179.73 (13)
C1—N1—C11—C16	151.11 (12)	C13—C14—C15—C16	−0.1 (2)
C1—N1—C11—C12	−33.70 (18)	C12—C11—C16—C15	−0.27 (18)
C16—C11—C12—C13	0.14 (18)	N1—C11—C16—C15	175.08 (11)
N1—C11—C12—C13	−174.99 (11)	C14—C15—C16—C11	0.2 (2)
C11—C12—C13—C14	0.03 (19)		

### Hydrogen-bond geometry (Å, °)

**Table:**

*D*—H···*A*	*D*—H	H···*A*	*D*···*A*	*D*—H···*A*
N1—H71···O1^i^	0.861 (18)	2.009 (18)	2.8643 (13)	172.1 (15)
C2—H2B···O1^i^	0.99	2.55	3.4203 (15)	147.

Symmetry codes: (i) *x*+1/2, *y*, −*z*+1/2.

## References

[bb1] Akkurt, M., Yalçın, Ş. P., Türkmen, H. & Büyükgüngör, O. (2010). *Acta Cryst.* E**66**, o1559–o1560. 10.1107/S1600536810020465 PMC3006765 21587803

[bb2] Bernstein, J., Davis, R. E., Shimoni, L. & Chang, N.-L. (1995). *Angew. Chem. Int. Ed. Engl.* **34**, 1555–1573.

[bb3] Betz, R., McCleland, C. & Marchand, H. (2011). *Acta Cryst.* E**67**, o1207. 10.1107/S1600536811014644 PMC3089288 21754506

[bb4] Bruker (2008). *SADABS* Bruker Inc., Madison, Wisconsin, USA.

[bb5] Bruker (2010). *APEX2* and *SAINT* Bruker AXS Inc., Madison, USA.

[bb6] Etter, M. C., MacDonald, J. C. & Bernstein, J. (1990). *Acta Cryst.* B**46**, 256–262. 10.1107/s0108768189012929 2344397

[bb7] Farrugia, L. J. (1997). *J. Appl. Cryst.* **30**, 565.

[bb8] Huang, J.-Y. & Xu, W. (2006). *Acta Cryst.* E**62**, o2651–o2652.

[bb9] Macrae, C. F., Bruno, I. J., Chisholm, J. A., Edgington, P. R., McCabe, P., Pidcock, E., Rodriguez-Monge, L., Taylor, R., van de Streek, J. & Wood, P. A. (2008). *J. Appl. Cryst.* **41**, 466–470.

[bb10] Mijin, D. & Marinkovic, A. (2006). *Synth. Commun.* **36**, 193–198.

[bb11] Mijin, D. Z., Prascevic, M. & Petrovic, S. D. (2008). *J. Serb. Chem. Soc.* **73**, 945–950.

[bb12] Moreno-Fuquen, R., Quintero, D. E., Zuluaga, F., Haiduke, R. L. A. & Kennedy, A. R. (2011). *Acta Cryst.* E**67**, o659. 10.1107/S1600536811005320 PMC3051943 21522410

[bb13] Praveen, A. S., Jasinski, J. P., Golen, J. A., Narayana, B. & Yathirajan, H. S. (2011). *Acta Cryst.* E**67**, o1826. 10.1107/S1600536811024597 PMC3151961 21837194

[bb14] Sheldrick, G. M. (2008). *Acta Cryst.* A**64**, 112–122. 10.1107/S0108767307043930 18156677

[bb15] Spek, A. L. (2009). *Acta Cryst.* D**65**, 148–155. 10.1107/S090744490804362X PMC2631630 19171970

[bb16] Wu, W.-N., Cheng, F.-X., Yan, L. & Tang, N. (2008). *J. Coord. Chem.* **61**, 2207–2215.

[bb17] Wu, W.-N., Wang, Y., Zhang, A.-Y., Zhao, R.-Q. & Wang, Q.-F. (2010). *Acta Cryst.* E**66**, m288. 10.1107/S160053681000471X PMC2983540 21580233

